# Comparison of Health Awareness in South Korean Middle School Students According to Type of Online Physical Education Classes during the COVID-19 Pandemic

**DOI:** 10.3390/ijerph18157937

**Published:** 2021-07-27

**Authors:** Jung-In Yoo, Joung-Kyue Han, Hyun-Su Youn, Joo-Hyug Jung

**Affiliations:** 1 Department of Physical Education, College of Education, Korea University, Seoul 02841, Korea; surfyji@korea.ac.kr; 2 College of Sport Sciences, Chung-Ang University, Anseong 17546, Korea; jkhan@cau.ac.kr; 3 Department of Physical Education, College of Education, WonKwang University, Iksan 54538, Korea

**Keywords:** middle school students, health perception, importance–performance analysis (IPA), online physical education class types, COVID-19

## Abstract

Coronavirus disease 2019 popularized online classes to prevent educational deficits affected by the pandemic. This study aimed to assess the differences in the importance and performance of health awareness in Korean middle school students according to the types of online physical education classes they attended during the coronavirus disease 2019 pandemic. Overall, 583 participants were selected using a convenience sampling method; the data were obtained through an online survey using Google forms. Frequency analysis, reliability analysis, independent sample *t*-test, and importance–performance analysis were performed. First, the differences between importance and performance were found to be the most for sleep and physical activity management, and the least for disease and hygiene management. In addition, both groups demonstrated higher importance and performance for hygiene and disease management. There were significant differences in the importance and performance of all the sub-factors. Second, hygiene, disease management, and mental health management were found in quadrant I in both groups, while physical activity, sleep, and dietary habit management were in quadrant III. No factors were in quadrants II and IV. Correspondingly, there was no significant difference in adolescents’ health awareness between the assignment-based online group and interactive online group.

## 1. Introduction

Coronavirus disease 2019 (COVID-19) is a respiratory infection that has caused a global pandemic. Consequently, the World Health Organization (WHO) declared it an “international public health emergency” in 2020, making it the third pandemic following the Hong Kong (1968) and swine flus (2009) [1]. Its global spread has significantly changed everyone’s lives in various cultural, economic, and societal aspects. Among many alterations, a new trend, “untact,” has emerged following COVID-19 measures, including social distancing and working from home, in order to prevent the spread of the infection.

The “untact” culture of performing everyday tasks in non-face-to-face online settings has been accelerated by the pandemic. In particular, the “ontact” era, which refers to online connections to the outside world in “untact” settings, has encouraged virtual sports, concerts, and lectures as well as innovative developments such as video conferences, online financing, and online medical appointments. Such changes have also affected academia. In Korea, after postponing commencement in 2020, schools officially began virtually in April; moreover, the increase in the number of non-face-to-face learners has become a representative example of “ontact”.

Online classes require extensive preparation by schools. New tasks for remote virtual lectures have been assigned to teachers, and novel measures have been established for practical online teaching and learning, from creating online platforms to implementing remote lectures. Teachers have agreed that online classes are a means to prevent educational deficits, and have actively prepared for remote online lectures, although the learning effects may not be the same.

The same measures have been applied for physical education (PE) classes; however, given that their focus is on physical activities, the challenges experienced are different from other subjects. Teachers who teach online have expressed difficulties in implementing virtual lectures, where the value of PE is unverified. They have experienced complications in designing curriculum plans and teaching-learning environments. In addition, applying the teaching methods, interacting with students, evaluating their performance, preparing classes, and conducting them every week have also been found to be problematic [2].

Recently, PE classes have been slowly stabilizing after the initial chaos caused due to COVID-19. Some PE teachers have devised interactive classes through real-time video instructions and distance education platforms to effectively communicate with their students. Furthermore, others have designed lectures that use recorded content to offer theoretical and practical learning. Additionally, task-oriented PE classes have also been implemented to evaluate student achievement. Such lectures involve assignments related to PE achievement standards; students are instructed to complete and upload their work, and teachers provide them with specific feedback through online chat rooms [3].

Although educators have considered alternatives that most effectively deliver the necessary contents, their students may doubt the learning effects of these online PE classes. During the COVID-19 pandemic, health problems such as corona blues, helplessness, and depression caused due to social distancing, a contactless culture, working from home, and quarantine have been observed in adults [4,5,6]. Furthermore, limited leisure, clubs, and sports activities have threatened the physical health of the general population [7,8,9]. It has been postulated that middle school students, who are physically more active than adults, are suffering as well in the limited environment of online classes [10,11,12].

One of the most important objectives of PE is “healthy development and encouragement of healthy lifestyles in students.” Good health is not only necessary for survival but is also a prerequisite for happiness. In general, healthy individuals have a normal physical function, are aware of the body feeling rewarded and energetic, maintain a normal appetite and stable weight, sleep sufficiently, experience mental and physical stability, and find harmony in daily life; moreover, they have no diseases [13]. Therefore, the assessment of health awareness among middle school students attending online PE classes for over a year may be an index to gauge the effects of online PE classes on their learning during the COVID-19 pandemic. Additionally, these health indicators may provide important data that could indirectly evaluate happiness in middle school students. Health awareness is a subjective process of being conscious of external stimuli related to wellbeing through sensory organs [14,15]. Specifically, it is a relative concept that is perceived differently by each individual depending on their thoughts and interpretations of themselves [16].

Previous studies have analyzed the importance and performance of health, an important variable in our study. Izadi et al., Lopes and Maia, and Miranda et al. [17,18,19] assessed the significance and performance of health services, while Rau et al. [20] examined the importance of recording personal health. Furthermore, Lee et al. [21] investigated health awareness in adolescents. Essentially, importance–performance analysis (IPA) studies on health have mainly been conducted on patients and have been limited to specific subjects and situations. Additionally, none of the studies was carried out during a pandemic situation such as the COVID-19 disease outbreak.

In addition, a number of studies have been conducted on adolescents’ health during the COVID-19 pandemic. Specifically, studies were conducted on adolescents’ mental health during the pandemic, and most of the studies reported that adolescents’ mental health deteriorated under the pandemic. Similarly, previous studies revealed that COVID-19 increased depression, anxiety, social isolation, maladjustment, and stress in adolescents, and it deteriorated the physical health of adolescents [22,23,24,25,26]. COVID-19 was also found to have increased the number of obese adolescents [27], and decreased physical activity [28,29]. In addition, the health-related quality of life (HRQoL) of adolescents decreased [30] and their lifestyle deteriorated during the pandemic [21,31].

However, none of these previous studies addressed the issue of adolescents’ online physical education during the pandemic. In addition, there is inadequate research to compare and analyze the differences in importance and implementation of various aspects of health factors. Considering the limitations of these prior studies, the current study aimed to establish how importantly adolescents perceive and implement “health” in the context of the COVID-19 disease outbreak, which emerged suddenly. Further, we aimed to analyze the health awareness level of Korean teenagers with persistent learning about the value of health through online physical education classes.

This study divided middle school students into those who participated in interactive online PE classes and those who attended assignment-based PE classes. Their health awareness was analyzed and divided into the following sub-factors: the management of mental health, disease, physical activity, sleep, dietary habits, and hygiene. Moreover, an IPA was conducted for empirical analysis as the relationships among the importance and performance of health in middle school students, strategic priorities, and their differences in health awareness would be useful for evaluating online PE classes and designing future curricula. Additionally, the findings of this study serve as important basic data for future planning and implementation of health education in public and private educational institutions.

## 2. Materials and Methods

### 2.1. Participants

After obtaining approval from the Institutional Review Board (IRB) of the Wonkwang University (WKIRB-202009-SB-053), convenience sampling, a non-probability sampling method, was employed. In the analysis conducted using the survey studies, a sample size of 10 times or more per observed variable is required [32]. Considering the size of these survey questions (51EA), the sample size was set at 583. The 583 students who participated in the study were selected from the K and C middle schools and categorized into two groups—assignment-based and interactive online PE classes, respectively. Students at K middle school (*n* = 334) participated in the assignment-based online class and students at C middle school (*n* = 249) participated in the interactive-based online class. All the participants were in the first year of middle school and about 13 years old. They were requested to complete a Google form questionnaire. Their demographic characteristics are presented in Table 1.

### 2.2. Instruments

This study employed a nominal scale that comprised two items assessing the participants’ general characteristics and the type of online class attended. The tool “Health Perception Scale,” developed by Ware [16] was utilized in this study; its validity and reliability were verified by Lee, So, and Youn; Barakat et al.; Jones; and Lee et al. [21,33,34,35]. It was modified according to the purpose of the current study to examine health awareness. This measure consists of the following six factors: mental health, disease, physical activity, sleep, dietary habits, and hygiene management. Each of these items was evaluated individually on a five-point Likert scale ranging from “strongly agree” (5 points) to “strongly disagree” (1 point).

### 2.3. Reliability of Instruments

Cronbach’s alpha coefficient was calculated to verify the reliability and internal consistency of the scale used in the study; the results are shown in Table 2. The sub-domains of health awareness demonstrated coefficients that were higher than 0.70, ranging from 0.705 to 0.93, suggesting high inter-item consistency [36]. Additionally, the scale’s reliability increased after excluding the sub-factors having higher “alpha if item deleted” than “Cronbach’s alpha.” The research was conducted after deleting one question (sleep management, #4).

### 2.4. Procedure and Data Analysis

This study compared the health awareness differences between assignment-based online classes and interactive online classes for students who participated in both online and face-to-face classes in September and December 2020. In assignment-based online classes, a teacher loads class data on an online platform and students take the content statically, while in interactive online classes, teachers and students connect to the online platform at the same time and conduct two-way active classes together. Prior to the COVID-19 pandemic, the participants’ physical education class was face-to-face. The average number of students per class in both schools selected for the study was 30, and researchers contacted the school online prior to this study to explain the purpose of the study, and consent to participate in the study was thereafter obtained from teachers, students, and parents. The data were collected using a Google form questionnaire and analyzed using the SPSS software (version 18.0; IBM Corp., Armonk, NY, USA). The detailed analysis method was as follows: First, a frequency analysis was conducted to confirm the demographics of the participants. Second, Cronbach’s α was calculated to verify the tool’s reliability. Third, a paired sample *t*-test was carried out to assess health awareness and the differences between the importance and performance of each factor. Subsequently, an independent sample *t*-test was performed to examine the importance and performance of the factors between the two groups. Finally, an IPA was conducted to validate the importance and performance of each factor; a *p*-value less than 0.001 was considered significant.

## 3. Results

### 3.1. Differences in the Importance–Performance of Health Awareness According to the Types of Online PE Classes

Table 3 and Table 4 show the results of the paired sample *t*-tests, indicating significant differences between the importance and performance of all health awareness factors of the students who participated in the assignment-based and interactive online PE classes. Further, the independent sample *t*-test revealed no significant differences in any of the factors of health awareness between the two groups of students.

### 3.2. Analysis of Differences in Importance–Performance Matrix

**Figure 1:**
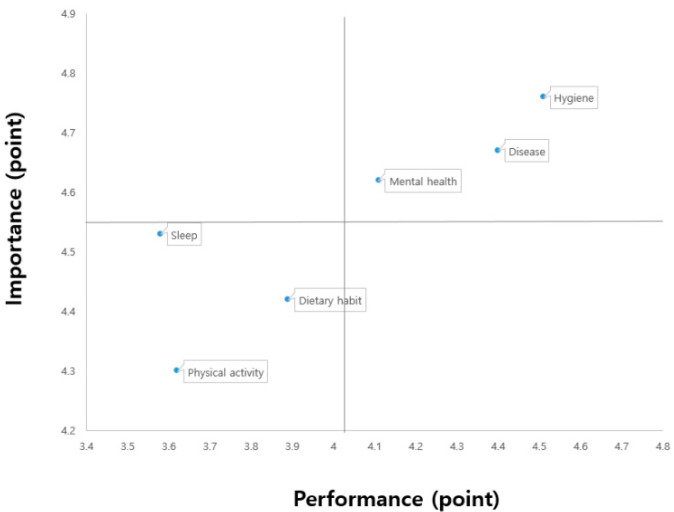
K middle school with assignment-based online PE classes.

**Figure 2:**
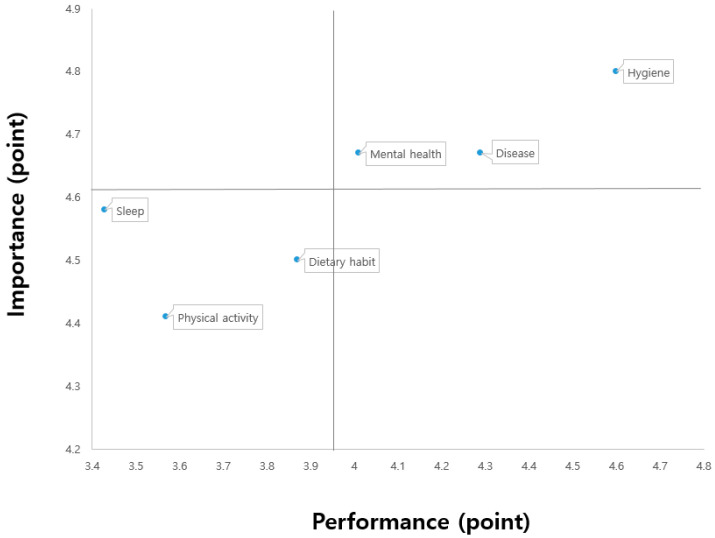
C middle school with interactive online PE classes.

To plot the IPA matrix, the students who participated in the assignment-based and interactive online PE classes were divided using the mean importance values of 4.55 and 4.61, respectively, and the mean performance values of 4.02 and 3.96, respectively. The results are shown in Figure 1 and Figure 2.

First, quadrant I (keep up the good work) indicated high importance and performance. Specifically, the students of both K and C middle schools had high performance in hygiene, disease, and mental health management. Second, quadrant II (concentrate here) revealed high importance and low performance, suggesting that urgent improvement was needed. No factor was found in quadrant II for either group. Third, quadrant III (low priority) demonstrated low importance and performance, signifying the requirement for extensive efforts. Specifically, for both groups, the importance and performance levels were low for physical activity, sleep, and dietary habit management. Fourth, quadrant IV (possible overkill) indicated low importance and high performance, suggesting that excessive effort was expended. In both groups, none of the factors belonged to this quadrant. Although there were slight differences in the location of the factors on the IPA matrix, the same results were observed in all four quadrants between the two groups of students. The sub-factors of health awareness among students at K middle school and C middle school located in the IPA grid were distributed as shown in Table 5.

## 4. Discussion

This study aimed to assess the importance and performance of health awareness among middle school students and evaluate the differences in health awareness between the students who participated in the assignment-based and interactive online PE classes.

First, the difference between the importance and performance of health awareness was the greatest for sleep and physical activity management, and the lowest for disease and hygiene management. In addition, both groups demonstrated higher importance and performance for hygiene and disease management. There were significant differences in the importance and performance of all the sub-factors. These results were obtained because of the successful K-quarantine implemented by the Korean government and continuous education in schools. The increase in quarantine policy and education by the government highlighted the significance of hygiene awareness, leading to personal hygiene practices among middle school students. In contrast, physical activity, sleep, and dietary habit management had low importance and performance in both groups. Our findings are similar to those of Henchoz, Cavalli, and Girardin [37], who reported that adolescents neglect their health practice behaviors despite the physical activity, sleep, and dietary habits being essential factors for the maintenance and promotion of health. It is also in line with the findings of Lee, So, and Youn [21] and Mastorci et al. [31], which showed a tendency of adolescents’ lifestyles deteriorating because of the COVID-19 pandemic. The outcomes of the current study indicated the students’ awareness of the importance of preventing and treating the infection as well as their relatively low interest in the management of other health factors, excluding hygiene, disease, and mental health. Following the COVID-19 pandemic, social distancing and restricted face-to-face classes significantly decreased their physical activity, and excessive exposure to digital media for studying and attending online classes may have caused difficulties in managing sleep and maintaining appropriate dietary habits.

Furthermore, the current education policy in Korea focuses on college entrance exams that already negatively affect the sleep and physical activity of adolescents; moreover, the COVID-19 pandemic may have further aggravated these factors. Therefore, efforts are primarily required for factors with significant differences between their importance and performance to lessen this gap. The happiness of Korean adolescents has previously been one of the lowest among the OECD (Organization for Economic Cooperation and Development) countries; additionally, corona blues, depression, and isolation during the COVID-19 pandemic may have worsened their mental health [22,23,24,25,26].

Most importantly, physical activity had the lowest importance and performance in both groups, suggesting that further efforts are required for its improvement. Globally, researchers have warned that COVID-19 pandemic may lead to an increased risk of obesity due to limited outdoor activities and face-to-face interactions in school [27]). Although non-face-to-face classes have been conducted for students, the level of activity is similar to that during vacations. Prior to the pandemic, students participated in PE classes and various other activities for physical activity. Approximately 79% of students indicated positive responses to school activities according to the 2016 School Sports Activity Participation Satisfaction Survey by the Korean Institute for Curriculum Evaluation [38]. However, the current situation restricts access to public playgrounds and group sports that may have contributed to decreased awareness of physical activity.

Second, hygiene, disease, and mental health management were shown in quadrant I (keep up the good work) in both the student groups, while physical activity, sleep, and dietary habit management were in quadrant III (low priority). However, none of the factors were located in quadrants II (concentrate here) or IV (possible overkill), suggesting that there was no difference in health awareness between the two groups. In a previous study on the importance and performance of health awareness in adolescents, Lee, So, and Youn [21] demonstrated that hygiene and disease management were in quadrant I, while mental health, physical activity, sleep, and dietary habit management were in quadrants II and III, respectively. These findings support the results of the present study.

To overcome the COVID-19 pandemic, many PE teachers introduced home training and interactive curriculum content to encourage students’ physical activity. However, as revealed in this study, interactive online PE classes have limitations in increasing the awareness of physical activity management in middle school students. This suggests that although educators may feel that the level of physical activity provided through interactive online classes is sufficient, its awareness in students may not be determined by merely the number of activities. Physical activities for middle school students may include those on the grounds or gyms and group activities with friends and partners. The lack of difference in the IPA between assignment-based and interactive online PE classes supports this possibility. Thus, the COVID-19 pandemic led to online PE classes as well as limited school sports clubs and after-school activities. Additionally, Saturday sports day and various extracurricular and leisure activities were restricted, resulting in decreased awareness and performance of physical activities. This is in line with the findings of Lee, So, and Lee [28] and Bronikowska et al. [29], which revealed that physical activity levels decreased due to the effects of COVID-19.

As shown by Lee et al., and Bae and Hyeon [24,39], who reported the relationship between physical activity participation and health awareness, it is necessary to encourage physical activity during pandemics to promote adequate physical development and immunity in middle school students [21]. Intrinsically, various PE researchers have developed and implemented indoor activities that can be performed at home as the pandemic is likely to last long. However, as indicated in this study, other types of activities may also be necessary to promote health awareness, particularly physical activities, among middle school students. For example, both face-to-face and non-face-to-face classes have been conducted simultaneously in Korea during the COVID-19 pandemic. There may be differences in the number of school days attended; however, it is believed that the blended curriculum involving both types of classes will continue in the near future. Thus, in this process, it would be essential to improve the curriculum contents to select courses that are knowledge-based and can be conducted online; moreover, other lectures that involve experiments and practice, such as PE classes, must be provided through offline meetings. Overall, the school curriculum needs to be revised to reflect the characteristics of each subject and offer a combination of face-to-face and non-face-to-face sessions.

Finally, based on the limitations of the current study, suggestions for follow-up studies are as follows: First, the current study assessed differences in health awareness according to the types of online PE classes. However, future studies should verify these differences between the two types of online classes. Second, this research only included middle school students from South Korea. In future studies, participants of different ages from various countries and regions should be recruited. Lastly, this study employed IPA, which is a quantitative research method, to evaluate health awareness among middle school students. In the future, both quantitative and qualitative methods will be necessary to examine health awareness in both teachers and students.

## 5. Conclusions

In this study, the conclusions of the comparative analysis of health awareness of the types of online PE class between the two middle school groups were as follows. Hygiene, disease, and mental health management were shown in quadrant I in both groups, while physical activity, sleep, and dietary habit management were in quadrant III. However, no factors were located in quadrants II and IV. Correspondingly, there was no significant difference in adolescents’ health awareness between the assignment-based online group and interactive online group. Therefore, in addition to the development of various interactive online PE programs, revised curricula and active efforts of teachers are required to promote physical activities such as face-to-face PE classes, school club events, and after-school sports activities.

## Figures and Tables

**Table 1:** Demographics of the study participants.

Characteristics	Classification	Frequency	%
Sex	Men	272	46.7
	Women	311	53.3
Class Type	Assignment-based online class (K middle school)	334	57.3
	Interactive online class (C middle school)	249	42.7
Total		583	100

**Table 2:** Results of the reliability analysis.

Factors		Cronbach’s α
Mental health management	Importance	0.895
	Performance	0.915
Disease management	Importance	0.807
	Performance	0.705
Physical activity management	Importance	0.877
	Performance	0.842
Sleep management	Importance	0.806
	Performance	0.769
Dietary habit management	Importance	0.843
	Performance	0.712
Hygiene management	Importance	0.931
	Performance	0.843

**Table 3:** Differences in the importance–performance of health awareness in K middle school students participating in the assignment-based online PE classes.

Factors	Importance		Performance		Difference in Mean	Rank	*t*	*p*
	M	SD	M	SD				
Mental health management	4.62	0.49	4.11	0.73	0.51	4	15.214	0.000 ***
Disease management	4.67	0.51	4.40	0.65	0.27	5	9.359	0.000 ***
Physical activity management	4.30	0.68	3.62	0.95	0.68	2	15.555	0.000 ***
Sleep management	4.53	0.60	3.58	0.97	0.95	1	19.478	0.000 ***
Dietary habit management	4.42	0.67	3.89	0.82	0.53	3	13.742	0.000 ***
Hygiene management	4.76	0.43	4.51	0.47	0.25	6	15.112	0.000 ***

*** *p* < 0.001, tested by paired sample *t*-test.

**Table 4:** Differences in the importance–performance of health awareness in the C middle school students participating in the interactive PE classes.

Factors	Importance		Performance		Difference in Mean	Rank	*t*	*p*
	M	SD	M	SD				
Mental health management	4.67	0.52	4.01	0.81	0.66	3	14.504	0.000 ***
Disease management	4.67	0.54	4.29	0.70	0.38	5	11.570	0.000 ***
Physical activity management	4.41	0.71	3.57	0.99	0.84	2	14.210	0.000 ***
Sleep management	4.58	0.62	3.43	1.00	1.15	1	18.561	0.000 ***
Dietary habit management	4.50	0.67	3.87	0.80	0.63	4	14.523	0.000 ***
Hygiene management	4.80	0.48	4.60	0.51	0.20	6	12.399	0.000 ***

*** *p* < 0.001, tested by paired sample *t*-test.

**Table 5:** Distribution of health awareness factors in the K and C middle school students.

Classification	Standard	School name	Factors
Quadrant I (keep up the good work)	importance ↑, performance ↑	K middle school	Hygiene, disease, and mental health management
		C middle school	Hygiene, disease, and mental health management
Quadrant II (concentrate here)	importance ↑, performance ↓	K middle school	None
		C middle school	None
Quadrant III (low priority)	importance ↓, performance ↓	K middle school	Physical activity, sleep, and dietary habit management
		C middle school	Physical activity, sleep, and dietary habit management
Quadrant IV (possible overkill)	importance ↓, performance ↑	K middle school	None
		C middle school	None

K middle school = Assignment-based online class, C middle school = Interactive-based online class.

## Data Availability

The data presented in this study are available on request to the authors.
